# Changes in Physical Activity Patterns from Childhood to Adolescence: Genobox Longitudinal Study

**DOI:** 10.3390/ijerph17197227

**Published:** 2020-10-02

**Authors:** Francisco Jesús Llorente-Cantarero, Francisco Javier Aguilar-Gómez, Augusto Anguita-Ruiz, Azahara Iris Rupérez, Rocío Vázquez-Cobela, Katherine Flores-Rojas, Concepción M. Aguilera, Esther M. Gonzalez-Gil, Mercedes Gil-Campos, Gloria Bueno-Lozano, Rosaura Leis

**Affiliations:** 1 Department of Specific Didactics, Faculty of Education, University of Córdoba, 14071 Córdoba, Spain; llorentefj@yahoo.es; 2 CIBEROBN, (Physiopathology of Obesity and Nutrition) Institute of Health Carlos III (ISCIII), 28029 Madrid, Spain; augustoanguitaruiz@gmail.com (A.A.-R.); airuperez@unizar.es (A.I.R.); caguiler@ugr.es (C.M.A.); esthergg@unizar.es (E.M.G.-G.); gbuenoloz@yahoo.es (G.B.-L.); mariarosaura.leis@usc.es (R.L.); 3 Metabolism and Investigation Unit, Reina Sofia University Hospital, Institute Maimónides of Biomedicine Investigation of Córdoba (IMIBIC), University of Córdoba, 14004 Córdoba, Spain; fjaviagui1992@gmail.com (F.J.A.-G.); katherine1.flores@gmail.com (K.F.-R.); 4 Department of Biochemistry and Molecular Biology II, Institute of Nutrition and Food Technology “José Mataix”, Center of Biomedical Research, University of Granada, Armilla, 18016 Granada, Spain; 5 Instituto de Investigación Biosanitaria IBS, 18012 Granada, Spain; 6 GENUD Researchgroup, University of Zaragoza, Instituto Agroalimentario de Aragón (IA2), Instituto de Investigación Sanitaria (IIS), 50009 Aragón, Zaragoza, Spain; 7 Unit of Investigation in Nutrition, Growth and Human Development of Galicia, Department of Pediatrics, University Clinical Hospital of Santiago, University of Santiago de Compostela, 15701 Santiago de Compostela, Spain; cobela.rocio@gmail.com; 8 Pediatric Endocrinology Unit, Lozano Blesa University Clinical Hospital of Zaragoza, Faculty of Medicine, University of Zaragoza, 50009 Zaragoza, Spain

**Keywords:** childhood, obesity, physical activity, pubertal status, sedentary time

## Abstract

Longitudinal changes of physical activity (PA) from childhood into adolescence have not been accurately described yet for the Spanish population. The aim of this study is to evaluate the changes of PA, assessed by accelerometry and anthropometric measures in a cohort of 213 children from the prepubertal to pubertal period, focusing on those with valid data from both time points (*n* = 75). Sedentary time (ST) increased about 50%, while all PA intensities declined from the pre-pubertal to pubertal period. Light PA (LPA) was the major contributor, decreasing by about 30%. Boys were more active than girls in both periods, but they showed a higher decline in PA, especially moderate-to-vigorous PA (MVPA). The proportion who reached the recommendation of 60 min of MVPA decreased by 33.3% in boys and 4.6% in girls. Children with obesity or overweight had lower MVPA than those with normal-weight in the pre-pubertal period, but no differences were found in the pubertal period. This study shows a decrease of PA and an increase of sedentarism in the transition from childhood to adolescence, particularly in boys. Regardless of body weight, adolescents tend to be less active. Therefore, prevention programs should be implemented to achieve optimal PA and reduce sedentarism during infancy considering the differences found by sex.

## 1. Introduction

Physical activity (PA) improves cardiorespiratory fitness and strengthens the musculoskeletal system, contributing to maintain an adequate body composition and preventing childhood obesity [1,2].

In this context, the World Health Organization (WHO) and other entities [3,4,5,6] have recommended at least 60 min per day of moderate-to-vigorous physical activity (MVPA) for children and adolescents [7,8]. A recent systematic review also highlighted the potential benefits of total PA and light PA (LPA), especially in the improvement of cardio-metabolic biomarkers [9].

On the other hand, sedentarism has been proposed as an independent risk factor of unhealthy outcomes, such as overweight or obesity, especially in adults. However, evidence in youth is less conclusive to date [10,11]. Sedentary behavior is characterized by a very low energy expenditure (≤1.5 of metabolic equivalents of task (METS)) in a sitting, reclining, or lying posture [12]. However, there are no recommendations for sedentary time (ST) but a suggestion to limit screen time (a component of ST) to no more than 2 h per day. A recent review revealed that less than 50% of European children and adolescents meet the WHO recommendations regarding PA when measured subjectively [13], but even those who achieve the 60 min of MVPA may also spend a high proportion of their time being sedentary [14].

In addition to these findings, the practice of PA seems to decrease progressively during childhood and adolescence, coupled to the increase of ST [15]. Although the reduction of MVPA has always received attention, the latest research has focused also on the importance of LPA decline [9,15]. There are several factors associated with the PA decline: biological, psychosocial, and environmental. Specifically, the influence of gender, pubertal status, or body mass index (BMI) have not been accurately described yet. Moreover, most of these studies include self-reported PA and/or a cross-sectional design [16,17,18,19], being few those with longitudinal data.

During childhood and adolescence, boys seem to perform more PA than girls [13], although the rate of decline by gender varies between studies especially related with social factors and others. This reduction in adolescents seems to occur earlier in girls (9–12 years) and later in boys (13–16 years) [17,20], suggesting that it may be related with pubertal status more than with chronological age. It seems that young people become less physically active as they progress along the maturation process [16,17,18,19]; thus, puberty could be a critical lapse for PA [21]. The appearance of secondary sexual characteristics, the changes in body composition, hormonal imbalance, and self-perception are related to the practice of PA in boys and girls [22]. So, puberty timing (e.g., the age of menarche or peak of high velocity) related to age and gender may be relevant in explaining the decrease in the practice of exercise.

A recent review [23] revealed that the practice of MVPA is significantly lower in children and adolescents with obesity than in their normal-weight peers, although differences are relatively small and both groups are below the recommendations. Moreover, no differences have been found in ST between BMI groups, without any information regarding total PA or LPA. The relationships between changes in body composition, gender, or puberty and PA, remain under investigation. This is of special importance for addressing population-based interventions.

Based on these previous observations, it is important to describe changes of PA and ST according to the presence or absence of pubertal development, as well as to corroborate if the differences previously reported in other countries, such as United Kingdom [15], between genders and BMI groups remain similar for the Spanish population. The aim of the present study is to analyze the time spent on all intensities of PA, measured by accelerometry, and ST in a cohort of children followed from pre-pubertal to pubertal status, focusing also on gender differences and BMI changes.

## 2. Materials and Methods

### 2.1. The Cross-Sectional Study Design

The present study was carried out under the framework of the GENOBOX study [24,25]. GENOBOX is a cross-sectional case-control, multicentre study carried out in children from 2012–2015. After assessing them in a first visit at the primary care centre, the children fulfilling the inclusion criteria and their parents were invited to the Endocrine Departments of the Reina Sofía University Hospital in Córdoba, University Clinical Hospital in Santiago de Compostela, and Lozano Blesa University Clinical Hospital in Zaragoza, obtaining a similar sample distribution among three regions.

Nine hundred and fifty-three prepubertal children were assessed based in the sample size estimation for the GENOBOX study [24,25]. Out of them, a subsample of 213 (27 from Córdoba, 104 from Santiago de Compostela, and 82 of Zaragoza) children (105 boys) was selected based on the following inclusion criteria for the present study: to have valid blood samples including sex hormones (follicle-stimulating hormone, luteinizing hormone, testosterone in boys, and estradiol in girls); being aged between 5–14 years and being in a pre-pubertal stage (Tanner I confirmed with sex hormones: follicle-stimulating hormone (<5.0 U/L), luteinizing hormone (<8 U/L), testosterone in boys (<0.5 ng/mL), and ostradiol (<10 pg/mL) in girls) at baseline, with an absence of endogenous obesity and metabolic diseases at recruitment, no use of medications for controlling blood pressure (BP), glucose, or lipid metabolism levels, and valid data for the present study variables; especially, with data from an accelerometer according to the protocol.

### 2.2. The Longitudinal Study Design

Two measurements were conducted on the selected children before and after the onset of puberty, being all of them part of the previously mentioned cross-sectional study population. All these children were first recruited as prepubertal children during the year period (2012–2015), baseline, and called again for follow-up medical consultation in 2018. All subjects with clinical signs of puberty at follow-up (at least Tanner II, confirmed with sex hormones), were included in the longitudinal study. Finally, 75 children presented valid data of PA, measured by accelerometers, at both prepubertal and pubertal stage. During the whole course of the study (2012–2018), children remained under regular medical monitoring by the same pediatricians.

Children and parents or custody holders were informed about the purpose and procedures of the study, giving the children their assent to participate. Signed written consents were obtained from the parents after the Ethics Committees of all participating institutions approved the study. We complied with the Declaration of Helsinki [26] and followed the recommendations of the Good Clinical Practice of the CEE (Central and Eastern Europe) (Document 111/3976/88 July 1990) and the legal, in-force Spanish regulation, which regulates Clinical Investigations in human beings (RD 223/04 on Clinical Assays).

### 2.3. Anthropometric and Clinical Measurements

Medical history and a physical examination including the evaluation of sexual maturity according to Tanner’s five-stage scale [27] were assessed in both visits, at prepubertal and pubertal stages, and confirmed with sexual hormone measurements. Anthropometric measurements were taken by a single examiner within each hospital. Body weight was measured using a standard beam balance. Height was measured using a precision stadiometer. Waist circumference (WC) was measured in fasting state by applying an inelastic tape horizontally midway between the lowest rib margin and the iliac crest of the standing child at the end of a gentle expiration. BMI was calculated (kg/m^2^), and overweight and obesity were defined using age and sex-specific BMI cut-off points of the International Obesity Task Force, equivalent to adult values of 25 kg/m^2^ for overweight and 30 kg/m^2^ for obesity [28]. In this study, three BMI groups were created to test differences in PA between them in the two time points (baseline and follow-up): normal-weight (NW), overweight (OW), and with obesity (OB). For the analysis of the changes in PA between the two time points, BMI-change groups were created as follows: NW-no change group, OW/OB-no change group, improving-BMI group (for those who changed from OB to OW or NW, and from OW to NW), and worsening-BMI group (for those who changed from NW to OW or OB, and from OW to OB).

Systolic and diastolic blood pressure (BP) were measured three times by the same examiner using an electronic manometer (Omrom, M6 AC) and following international recommendations [29], and the mean of the three measurements was considered the current value.

### 2.4. Biochemical Analysis

Blood samples were drawn from the antecubital vein between 08:00 and 09:30 h after an overnight fast. Routine blood tests were analyzed at the general laboratory of each participating hospital. Glucose (CV = 1.0%) was analyzed using the glucose oxidase method in an automatic analyzer (Roche-Hitachi Modular P and D Autoanalyzer; Roche Laboratory Systems, Mannheim, Germany), and plasma insulin was analyzed by radioimmunoassay (RIA) (CV = 2.6%) using an automatic microparticle analyzer (AxSYM; Abbott Laboratories, Abbott Park, IL, USA). Insulin resistance (IR) was calculated by the homeostatic model assessment of IR (HOMA-IR). Serum triacylglycerols (TAG) (CV = 1.5%), total cholesterol (CV = 0.9%), high density lipoprotein cholesterol (HDL-c) (CV = 0.8%), and low-density lipoprotein cholesterol (LDL-c) (CV = 1.5%) were measured using an automatic analyzer (Roche-Hitachi Modular P and D Autoanalyzer; Roche Laboratory Systems, Mannheim, Germany). The sex hormones follicle-stimulant hormone (FSH) (CV = 3.6%); luteinizing hormone (LH) (CV = 3.1%), testosterone (CV = 2%), and estradiol (CV = 1.8%) were measured by chemiluminescence using an automatic analyzer (Architec I4000, Abbott Laboratories, Abbott Park, IL, USA).

### 2.5. Accelerometry

ActiGraph GT3X+ accelerometers (ActiGraph; Pensacola, FL, USA) were used to assess PA levels in this study. Accelerometers were placed over the right iliac crest and held in place using an adjustable elastic belt for 24 h a day and could be removed only to shower or for nocturnal rest (if the instrument caused discomfort in sleeping). It was programmed for 15 epochs (period of 15 s), as previously recommended [30].

Accelerometry data were processed using the Actilife v6.13.3 program. Two rules were used for excluding data: (a) all negative counts were replaced by a missing data code, and (b) periods of 20 min or more of consecutive zero counts were replaced by a missing data code prior to further analysis, as recommended by Treuth et al. [31]. The output generated by the ActiGraph GT3X+ included the total volume of PA and each PA intensity as defined by the cut-points of the following counts per minute (CPMs) based in Evenson et al. [32] classification: sedentary: ≤100 CPM, light (LPA): >100–<2296 CPM, moderate (MPA): >2296–<4012 CPM, and vigorous PA (VPA): ≥4012 CPM. A minimum of 8 h of monitoring per day for at least 3 days including at least 1 weekend day was considered acceptable for the evaluation of PA and sedentary time.

After meeting these conditions, differences in time measured between the two timepoints may have been different and over- or underestimated in absolute values, so relative values of each PA intensity were calculated as follows: % of LPA = (min of LPA measured/min of total time measured) × 100, as previously [33].

### 2.6. Statistical Analyses

All continuous variables were tested for normality using the Kolmogorov test, and all were transformed through natural log, or square root or rank-based inverse normal transformation. Heteroscedasticity between groups was explored with the Levene test. Differences in the characteristics of the participants for prepubertal and pubertal periods were tested using Chi-square or t-paired tests.

In the cross-sectional study, the two-way ANOVA and Kruskal–Wallis were employed to assess group differences in the measurements according to standard statistical assumptions. In addition, the Dunn tests were applied conveniently as post-hoc analyses adjusted by age to determine which experimental groups differed from each other for the ANOVA. A *p*-value ≤ 0.05 was considered significant.

In the longitudinal study, mean (SD) differences in the time of ST/PA (all intensities), between the two time points were assessed for all subjects and separately by gender, BMI groups, and BMI-change groups, using paired t-tests, paired Wilcoxon signed rank tests, and Dunn tests conveniently, adjusted by age. Absolute values would overestimate the differences between prepubertal and pubertal time; thus, relative values of each level of PA were also calculated (min of intensity level of PA with regard to the total measured time, expressed as percentage).

On the other hand, differences between prepubertal and pubertal stage (∆) were calculated for BMI z-score and the PA variables. After that, given the co-linearity found between PA variables, several multivariable regression tests selecting changes in BMI-Z score as dependent variable, and changes in the different physical activity intensities as independent variables, as well as age, Tanner status, and gender were included in the model carried out; (Supplementary Table S1). A *p*-value ≤ 0.05 was considered as significant. All statistical procedures were conducted by using SPSS (IBM SPSS Statistics for MacOS, Version 25.0. Armonk, NY, USA).

## 3. Results

**Figure 1:**
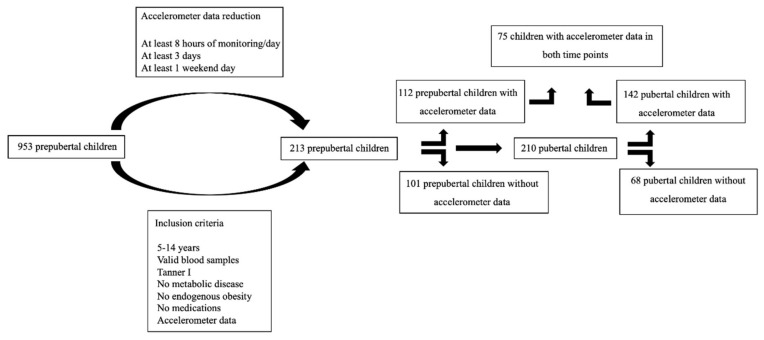
Design of the study.

Measurements of PA with an Actigraph device were collected from 52.8% of children at baseline (*n* = 112), 67.6% of children at follow-up (*n* = 142), and 35.2% of children at both time points (prepubertal and pubertal stages) (*n* = 75) (Figure 1). Table 1 shows the characteristics of the population in the group with both measurements. The number of days with valid PA data recorded was lower in prepubertal time than in pubertal time, although both of them were above the recommendations. The proportion of girls was a little higher, and it remained around 50% for each BMI group (data not shown). At baseline, 69% were children with overweight and obesity. The mean of the BMIZ-score in prepubertal children showed no significant difference to that at pubertal stage. At the end of follow-up, about 3/4 of adolescents showed no BMI group changes and the others had an improvement to a normal weight, or a worsening to obesity.

Total min of PA detected by the accelerometer were significantly higher at the pubertal (891.1 ± 169.6 min) than at prepubertal stage (771.8 ± 79.4 min). In Table 2, PA is presented for both times as the mean of measured min/day and relative values of these measurements for both groups. Absolute and relative values of ST were significantly higher in pubertal time compared with basal time, with no gender differences. In the adolescents, average ST increased by 66.9 min. LPA, MPA, and MVPA were lower in pubertal measures than in prepubertal. At baseline, only MVPA shows statistical differences in absolute values (*p* = 0.03) and, MPA and MVPA for relative values (*p* = 0.027 and *p* = 0.025, respectively). At the pubertal stage, only VPA showed significant gender differences. The decline of MPA and MVPA from prepubertal to pubertal period was higher in boys than girls. In contrast, VPA in boys was the only PA intensity which increased in absolute values between times (1.6 min/day). 

As Table 3 shows, around 60% of boys accomplished the recommendation of 60 min/day of MVPA at prepubertal period, while only 28% of girls did. In pubertal period, the proportion of adolescents who met this recommendation decreased in both genders, however, the decline was greater for boys than girls (33.3% vs. 4.6%, respectively).

Table 4 shows PA data according to BMI groups. At baseline, there were no differences in ST between BMI groups. NW children showed higher MPA, VPA, and MVPA values than OW and OB children. In fact, only NW prepubertal children reached 60 min of MVPA. At the pubertal stage, there were no differences between BMI groups for ST or any PA intensity. As seen previously, ST increased about 16% in relative values for all BMI groups, with a decline in PA, especially in LPA. This reduction in LPA, MPA, VPA, and MPVA tended to be higher for NW children than OW and OB children, with a tendency of similarity between BMI groups in the pubertal period.

Table 5 shows the PA measurements from the longitudinal analysis according to BMI-change groups. Subjects who did not change their BMI increased their ST for up to 70% of the time measured (about 15% more in relative values), which means about 1 h more per day of ST in pubertal stage. Those who improved their BMI had the highest increase in ST (about 18.8% in relative values). In contrast, those whose BMI worsened showed the shortest increase in ST (about 6%).

Regarding PA, the biggest increase in ST was replaced by a decline in LPA (about 88% of ST, which means about 35–40 min less per day), being statistically significant for all of them, except the worsening BMI group. The improving and not-changing BMI groups decreased MPA and MVPA in absolute and relative values, but VPA did not show differences in any of them. The worsening group did not show any difference between prepubertal and pubertal time in any group of PA intensities.

Finally, the results of the multivariable regression test are showed in the Table S1.

## 4. Discussion

Changes of PA and ST according to the presence or absence of pubertal development, as well as the differences between genders and BMI groups have been studied in this Spanish sample. The performance of PA in this Spanish cohort decreased from childhood to adolescence, being replaced by a rise in sedentarism. The time spent on all intensities of PA has been measured objectively by accelerometry, focused also on gender differences, and especially related with BMI changes.

This increase in ST which accounted for 72% of the total measured time in pubertal adolescents (Table 2), has been previously reported to be about 40 min per day than the baseline values, or reaching 90 min per day for British and North Americans [15,33,34,35]. Parallel to the increased ST, LPA was the main contributor in the reduction of PA, with a 14% decrease in relative values, while MPA, and especially VPA, remained stable (Table 2). Some researchers also found that the rise of ST matched the decrease in LPA in adolescence, while MVPA remained relatively constant during this stage [15,34,35,36]. Previous studies focused on MVPA as the most important contributor to the decrease of PA and its association with health benefits [1,20,37]. However, our data suggest that MVPA plays a small role in this reduction, at least in older children. It seems that VPA and MVPA levels were already low at the prepubertal time in our cohort, especially in girls, and only boys at baseline accomplished the 60 min of MVPA recommended by the WHO (Table 2). The latest research proposes an earlier decline of MVPA during early childhood [38,39,40], which makes us think about a stepped decline of the different PA intensities. We hypothesize that the decline of PA is produced from early childhood to adolescence in a staggered manner, with a decrease of MPA and VPA from early to late childhood, and a decline of LPA from late childhood to adolescence. ST increases progressively along this process. Most longitudinal studies are performed in adolescents or in late childhood populations [15,33,34,35,41], while early childhood data comes from cross-sectional and non-objective PA measures. Thus, future research in this area, especially about MVPA decline, should focus on this population.

In the present study, gender differences in PA were found for MPA, VPA, and MVPA, both in pre-pubertal and pubertal stages, showing that boys are more active than girls regardless of pubertal status. However, PA in boys decreased more prominently than in girls as in line with previous reports [33,41]. In our cohort, the proportion of boys who accomplished the 60 min recommendation fell from 60% to one quarter after puberty. In contrast, the percentage of girls who reached the WHO recommendation was already low at prepubertal time, as previously reported [40], showing a lighter decrease with adolescence (Table 3).

On the other hand, min of MVPA were higher in boys than girls in both time points, but differences tended to decrease over time, especially due to the reduction of activity in boys, similar to the previously reported results [33]. The gap between boys and girls in MPA seems also to be higher in the prepubertal than pubertal period, but it is lower in the prepubertal than pubertal period for VPA. In summary, differences in MPA tend to be similar, while those for VPA tend to increase.

PA and ST measurements between BMI groups showed higher MPA, VPA, and MVPA values in prepubertal NW children than in OW and OB, while no differences were found for the different PA intensities between groups classified by BMI in pubertal stage. Noteworthy, NW children were the only group who reached the 60 min recommendation of MVPA (Table 4). A recent systematic review [23] revealed that MVPA was significantly lower in children and adolescents with obesity compared to controls, but differences were small and none of the participants accomplished the WHO recommendation of MVPA. All BMI groups increased their ST and decreased their PA intensities in absolute and relative values, but the decrease of NW subjects was higher than those for OW and OB, showing that even these groups perform similar min of PA of any intensity in pubertal time, and there was a tendency of similarity between BMI groups from childhood to adolescence (Table 4).This may be because in the adolescence, children with obesity are more aware of their excess of weight and some of them try to compensate by improving their habits. So, perhaps the smallest increase in sedentary lifestyle is compensated with a greater interest in exercise than in the rest of the normal weight population. Most subjects in this study (around 70%) did not change their baseline BMI status after reaching puberty, and nearly 20% of them improved it (Table 1). These results could be explained because overweight/obesity children were addressed to the pediatric endocrinologist at prepubertal time where the received general dietary recommendations for weight management. The proportion of subjects with overweight and obesity at baseline in the population study was higher than in the general population. This also may be linked with the recruitment, which took place on the pediatric endocrinologist. However, PA levels did not vary when we studied normal-weight subjects separately.

Some interesting results were found when the sample was divided regarding their BMI changes. Subjects who improved or did not change their BMI increased their ST in relative values, at the expense of the decline of PA, especially LPA (Table 5). The worsening BMI group showed the shortest increase in ST (Table 5), although this could be explained by the reduced sample size. Similarly, VPA did not show differences in any of the groups.

Minutes measured of PA for prepubertal children were fewer than for pubertal, so relative values were calculated. This difference is related with the minutes that the Actigraph device was worn and the amount of them interpreted as null. Children probably tolerated this worse, and this reduced the valid time measured. We found differences in the total min of measured PA in previous studies without further explanation. Instead, Corder et al. [33,42] included several results as a percentage. Thus, differences in the proportion of PA levels relative to the time measured seem to be more informative than absolute mean differences.

The present study shows changes in objectively measured PA in prepubertal Spanish children who become adolescents. Most of the previous longitudinal data came from children or adolescents [15,33,34,41] and only a few studies have included children to follow until adolescence [40]. Subjects were classified regarding pubertal status instead of age, unlike previous literature [33,40]. This involved difficulties and losses in the follow-up, but allowed us to focus on the importance of the transition from childhood to adolescence in the decline of PA.

Previous studies used BMI as a static variable [33], so changes in PA behavior according to BMI and the bidirectional relationship between PA and weight status were difficult to interpret. This study brings a new approach with the inclusion of BMI as a dynamic variable, which allows to explore both changes in BMI and PA at the same time. However, a limitation to consider is that this subsample is from the GENOBOX study, so the proportion of subjects with overweight/obesity was higher than in the total population and trends of PA should be interpreted in that context. Moreover, in prepubertal time, the number of available devices for monitoring PA limited the sample size. As in pubertal time, some adolescents did not agree to wear the Actigraph device, and that restricted the sample size of the longitudinal group.

## 5. Conclusions

In conclusion, the results of this longitudinal study show a decrease of PA along with the increase of ST in the transition from childhood to adolescence, with differences by gender and BMI. Boys showed a higher decline in MVPA than girls, although remained more active. Regardless of body weight, teens tend to be less active. Therefore, it is necessary to implement measures at these stages to reduce sedentary lifestyle and at least maintain physical activity levels.

## Figures and Tables

**Table 1:** Characteristics of the longitudinal sample (n = 75) at prepubertal and pubertal periods and evolution in weight status.

	Prepubertal	Pubertal	p
Days of Physical activity	4.65 ± 0.70	6.52 ± 0.86	<000.1
Age (years)	8.46 ± 1.37	13.84 ± 1.88	<000.1
Females (%)	42 (56.0)		
BMI (Kg/m^2^)	21.92 ± 4.86	25.80 ± 6.78	0.001
BMI Z-Score	1.79 ± 1.95	1.63 ± 1.89	0.610
Cole groups (%)			
*Normal-weight*	23 (30.6)	29 (38.6)	
*Overweight*	19 (25.3)	18 (24.0)	
*With Obesity*	33 (44.0)	28 (37.3)	
Changes in BMI			
*No changes (%)*	55 (73.3)		
Normal-weight	21		
Overweight	9		
Obesity	25		
*Improvement (%)*	15 (20)		
Obesity to overweight	7		
Overweight to normal-weight	7		
Obesity to normal-weight	1		
*Worsening (%)*	5 (6.7)		
Normal-weight to overweight	2		
Overweight to obesity	3		
Normal-weight to obesity	0		

Data are expressed as mean ± DS. Differences between groups are presented in *p* column.

**Table 2:** Differences in physical activity levels between prepubertal and pubertal periods measured in mean minutes and relative percentages in the longitudinal sample (n = 75).

	Mean Values (min ± SD)				Relative Values (% ± SD)			
	Prepubertal	Pubertal	Δ (%)	*p*	Prepubertal	Pubertal	Δ (%)	*p*
**Sedentary**								
All	443.3 ± 74.1	636.8 ± 164.2	+43.6	<0.001	56.0 ± 6.7	71.1 ± 8.2	+15.1	<0.001
Boys	445.2 ± 78.1	661.9 ± 146.4	+48.6	<0.001	56.1 ± 6.3	71.6 ± 7.0	+15.5	<0.001
Girls	441.8 ± 71.6	617.1 ± 176.1	+39.6	<0.001	55.9 ± 7.0	70.7 ± 9.1	+14.8	<0.001
**Light PA**								
All	286.8 ± 47.8	200.6 ± 50.1	−32.0	<0.001	36.7 ± 5.6	23.4 ± 6.9	−13.3	<0.001
Boys	281.5 ± 43.7	202.7 ± 60.1	−27.9	<0.001	35.9 ± 4.8	22.5 ± 5.6	−13.4	<0.001
Girls	291.0 ± 50.8	199.0 ± 51.5	−31.6	<0.001	37.3 ± 6.2	24.1 ± 7.8	−13.2	<0.001
**Moderate PA**								
All	40.9 ± 13.3	31.1 ± 12.3	−23.9	<0.001	5.2 ± 1.6	3.6 ± 1.5	−1.6	<0.001
Boys	43.7 ± 14.8	31.7 ± 13.3	−27.4	<0.001	5.7 ± 1.7	3.6 ± 1.5	−2.1	<0.001
Girls	38.6 ± 11.7	30.6 ± 11.6	−20.7	0.003	4.9 ± 1.3 ^δ^	3.6 ± 1.5	−1.3 ^δ^	<0.001
**Vigorous PA**								
All	15.3 ± 9.2	15.2 ± 11.0	−0.6	0.965	1.9 ± 1.1	1.7 ± 1.2	−0.2	0.255
Boys	17.4 ± 10.5	19.0 ± 10.2	+9.1	0.379	2.1 ± 1.2	2.1 ± 1.3	0	0.809
Girls	13.6 ± 7.8	12.2 ± 8.9 ^λ^	−10.2 ^κ^	0.296	1.7 ± 0.9	1.4 ± 1.0 ^δ^	−0.3	0.139
**MVPA**								
All	56.1 ± 20.4	45.5 ± 18.0	−18.8	<0.001	7.1 ± 2.5	5.3 ± 2.3	−1.8	<0.001
Boys	61.4 ± 22.4	49.3 ± 18.9	−19.7	0.024	7.9 ± 2.8	5.6 ± 2.4	−2.3	0.001
Girls	52.1 ± 18.1 ^δ^	42.6 ± 16.9	−18.2	0.008	6.6 ± 2.1 ^δ^	5.1 ± 2.2	−1.5 ^δ^	0.001

PA: physical activity; MVPA: moderate-to-vigorous PA; Data of PA for both periods and variation between them are presented for total sample with absolute (mean) and relative values. Mean values are expressed as mean of min/day of each level of PA ± SD. Relative values are expressed as ((mean of min/day of any level of PA measured/mean of total min/day of PA measured) × 100) ± SD. Differences between sex are indicated in girls’ rows of “prepubertal”, “pubertal”, and “Δ” columns. Differences between periods for all, boys and/or girls are indicated in *p* column. Differences are expressed with: **δ** for *p* < 0.05; **λ** for *p* < 0.01; **κ** for *p* < 0.001.

**Table 3:** Distribution in percentage of children by gender in prepubertal and pubertal periods, related with moderate-to-vigorous physical activity in the longitudinal sample (*n* = 75).

	Boys (%)		Girls (%)	
**MVPA (min)**	**Prepubertal**	**Pubertal**	**Prepubertal**	**Pubertal**
<30	3 (9.0)	7 (21.2)	4 (9.5)	12 (28.5)
30–59.9	10 (30.3)	17 (51.5)	26 (61.9)	20 (47.6)
60–89.9	15 (45.4)	9 (27.2)	11 (26.1)	10 (23.8)
>90	5 (15.1)	0	1 (2.3)	0

**Table 4:** Comparison of physical activity levels and minutes of practice between prepubertal and pubertal periods according to BMI groups in the longitudinal sample (*n* = 75).

Total Sample (n = 75)	Mean Values				Relative Values			
	Prepubertal	Pubertal	Δ (%)	*p*	Prepubertal	Pubertal	Δ (%)	*p*
**Sedentary**								
NW	429.3 ± 63.1	641.3 ± 174.5 ^δ^	+49.3	<0.001	55.5 ± 7.3	73.2 ± 8.1	+17.7	<0.001
OW	428.1 ± 68.1	695.5 ± 120.5 ^λ^	+62.4	<0.001	56.5 ± 8.1	72.9 ± 5.7	+16.4	<0.001
OB	444.9 ± 85.0	624.9 ± 152.6	+40.4 ^κ^	<0.001	55.9 ± 6.4	71.1 ± 8.0	+15.2	<0.001
**Light PA**								
NW	279.5 ± 46.6	186.2 ± 52.5 ^δ^	−33.3 ^δ^	<0.001	36.1 ± 5.3	21.7 ± 6.7	−14.4	<0.001
OW	279.1 ± 52.9	208.4 ± 58.2	−25.3	<0.001	36.8 ± 6.6	21.9 ± 5.1	−14.9	<0.001
OB	290.1 ± 54.7	204.1 ± 56.0 ^δ^	−29.6	<0.001	37.1 ± 5.8	23.7 ± 6.4	−13.4	<0.001
**Moderate PA**								
NW	45.4 ± 13.6 ^κ^	27.6 ± 12 ^δ^	−39.2 ^κ^	<0.001	5.8 ± 1.6 ^κ^	3.2 ± 1.5	−2.6	<0.001
OW	36.7 ± 15.4	31.7 ± 12.5	−13.6	0.004	4.8 ± 1.9	3.3 ± 1.2	−1.5	<0.001
OB	39.7 ± 12.4 ^λ^	30.5 ± 14.0	−23.1 ^δ^	<0.001	5.1 ± 1.4 ^λ^	3.5 ± 1.5	−1.6	<0.001
**Vigorous PA**								
NW	18.8 ± 12.2 ^λ^	15.1 ± 10.2	−19.6 ^δ^	0.009	2.4 ± 1.4 ^κ^	1.7 ± 1.2	−0.7	<0.001
OW	13.1 ± 9.7	16.6 ± 11.9	+26.7 ^δ^	0.012	1.7 ± 1.1	1.7 ± 1.1	0	1.000
OB	14.2 ± 7.5 ^λ^	13.2 ± 10.8	−7.0	0.405	1.7 ± 0.9 ^κ^	1.5 ± 1.2	−0.2	0.143
**MVPA**								
NW	61.9 ± 21.2 ^λ^	42.0 ± 18.8	−32.1 ^δ^	<0.001	8.2 ± 2.9 ^κ^	4.9 ± 2.4	−3.3	<0.001
OW	49.8 ± 23.6	47.9 ± 19.9	−3.8	0.482	6.5 ± 2.8	5.0 ± 1.9	−1.5	<0.001
OB	54.8 ± 17.6 ^δ^	42.3 ± 19.7	−22.8	<0.001	6.9 ± 2.1 ^λ^	5.0 ± 2.4	−1.9	<0.001

PA: physical activity; MVPA: moderate-to-vigorous PA; NW: children normal-weight; OW: children with overweight; OB: children with obesity. Data of PA for each period and variation between them are presented with absolute (mean) and relative values. Mean values are expressed as mean of min/day of each level of PA ± SD. Relative values are expressed as ((mean of min/day of any level of PA measured/mean of total min/day of PA measured) × 100) ± SD. Differences between periods for NW, OW, and/or OB are indicated in Δ column. Differences between NW and OW are indicated in NW row. Differences between OW and OB are indicated in OW row. Differences between OB and NW are indicated in OB row. Differences are expressed with: **δ** for *p* < 0.05; **λ** for *p* < 0.01; **κ** for *p* < 0.001.

**Table 5:** Comparison of physical activity levels between prepubertal and pubertal periods according to BMI-change groups in the longitudinal sample (n = 75).

Total Sample (*n* = 75)	Mean Values				Relative Values			
	Prepubertal	Pubertal	Δ (%)	*p*	Prepubertal (%)	Pubertal (%)	Δ (%)	*p*
**Normal-Weight** **No Changes (*n* = 21)**								
Sedentary	423.0 ± 60.6	595.0 ± 183.1	+40.6	0.001	55.2 ± 6.3	69.9 ± 9.3	+14.7 ^β^	<0.001
Light	279.7 ± 42.0	196.2 ± 56.2	−29.8 ^β^	<0.001	3.6 ± 4.6	24.1 ± 7.7	−12.4	<0.001
Moderate	44.8 ± 10.5	30.5 ± 12.4	−31.9	0.001	5.8 ± 1.3	3.8 ± 1.7	−2.0 ^β^	0.001
Vigorous	17.9 ± 9.7	16.5 ± 10.0	−7.8	0.523	2.3 ± 1.2	2.0 ± 1.2	−0.2	0.384
MVPA	62.7 ± 19.2	47.0 ± 18.9	−25.0	0.007	8.2 ± 2.4	5.8 ± 2.6	−2.3	0.007
**Overweight/With Obesity** **No changes (*n* = 34)**								
Sedentary	445.8 ± 79.1	639.1 ± 162.1	+43.3	<0.001	55.4 ± 6.3	70.5 ± 7.4	+15.1 ^β^	<0.001
Light	300.7 ± 45.7	210.5 ± 51.8	−29.9 ^β^	<0.001	37.6 ± 6.2	24.0 ± 6.5	−13.5 ^β^	<0.001
Moderate	40.0 ± 12.4	32.2 ± 12.1	−19.5	0.016	5.0 ± 1.2	3.7 ± 1.3	−1.3	0.001
Vigorous	15.2 ± 9.6	14.9 ± 11.5	−1.9	0.885	1.8 ± 1.0	1.6 ± 1.1	−0.2	0.294
MVPA	56.3 ± 19.6	47.5 ± 18.8	−15.6	0.066	6.9 ± 2.2	5.3 ± 1.9	−1.6	0.005
**Improving (*n* = 15)**								
Sedentary	465.4 ± 70.4	728.6 ± 119.1	+56.5	<0.001	57.6 ± 5.4	76.5 ± 6.7	+18.8 ^β^	<0.001
Light	274.9 ± 44.2	178.1 ± 62.6	−35.2 ^β^	<0.001	35.8 ± 4.0	19.2 ± 6.2	−16.5 ^β^	<0.001
Moderate	37.5 ± 15.9	26.0 ± 10.0	−30.6	0.020	4.8 ± 2.1	2.6 ± 0.7	−2.1 ^β^	0.003
Vigorous	13.4 ± 7.8	13.9 ± 9.8	+3.7	0.813	1.6 ± 1.0	1.5 ± 1.1	−0.1	0.626
MVPA	50.6 ± 22.9	39.0 ± 15.9	−22.9	0.045	6.4 ± 3.0	4.1 ± 1.6	−2.2	0.010
**Worsening (*n* = 5)**								
Sedentary	444.6 ± 100.6	521.3 ± 85.3	+17.2	0.285	58.5 ± 12.8	64.4 ± 5.6	+5.8 ^δ,λ,κ^	0.329
Light	263.1 ± 78.7	226.1 ± 20.7	−14.0 ^δ,λ,κ^	0.354	34.5 ± 9.6	28.1 ± 2.8	−6.3 ^λ,κ^	0.252
Moderate	41.8 ± 20.8	43.1 ± 14.4	+3.1	0.834	5.4 ± 2.5	5.3 ± 1.7	−0.1 ^δ,κ^	0.932
Vigorous	10.4 ± 7.2	16.0 ± 16.8	+53.8	0.306	1.3 ± 0.9	1.9 ± 2.0	+0.5	0.383
MVPA	42.3 ± 16.3	46.5 ± 13.0	+9.9	0.630	6.8 ± 3.2	7.3 ± 3.7	+0.5	0.595

MVPA: moderate-to-vigorous physical activity; Improving: Subjects with overweight or obesity in prepubertal time and changed to overweight or normal-weight, respectively, in pubertal time. Worsening: Subjects who were normal-weight or overweight in prepubertal time and changed to overweight or obesity, respectively, in pubertal time. Data of PA and variation between periods are presented for total sample with absolute (mean) and relative values. Mean values are expressed as mean of min/day of each level of PA ± SD. Relative values are expressed as ((mean of min/day of any level of PA measured/mean of total min/day of PA measured) × 100) ± SD. Differences between BMI-change groups (*p* < 0.05) according to “Δ” for each level of PA intensity are expressed in “Δ” column. Differences regarding “Normal-weight no changes” group are expressed with **δ**; differences regarding “OW/OB no changes group” are expressed with **λ**; differences regarding “Improving group” are expressed with **κ**; differences regarding “Worsening” group are expressed with **β**.

## Supplementary Materials

The following are available online at https://www.mdpi.com/1660-4601/17/19/7227/s1, Table S1. Associations of ∆BMIz-score with with ∆physical activity in intensities, age, sex and pubertal stage during the follow up in Genobox study using multiple regression analysis.
