# Supplementary material for: Changes in Physical Activity Patterns from Childhood to Adolescence: Genobox Longitudinal Study
Source: Int J Environ Res Public Health. 2020 Oct 2;17(19):7227. doi: 10.3390/ijerph17197227 (PMC7579043; doi:10.3390/ijerph17197227)
Supplement: Supplementary file 1 [file ijerph-17-07227-s001.pdf]

Supplementary Materials:

Supplementary table 1. Associations of ΔBMIz-score with with Δphysical activity in intensities, age, sex and pubertal stage during the follow up in Genobox study using multiple regression analysis

Explanatory Variables	β	95% CI	β Stand	p–value (β)
Sedentary	0.000	-0.002, 0.001	-0.054	0.654
Gender	-0.835	-1.577, -0.093	-0.331	0.028
Age	0.075	-.0166, 0.317	0.110	0.537
Tanner	-0.049	-0.401, 0.303	-0.053	0.783
R^2	0.105			
Light PA	0.003	-0.002, 0.008	0.150	0.227
Gender	-0.834	-1.516, -0.152	-0.345	0.017
Age	0.095	-0.130, 0.321	0.147	0.402
Tanner	-0.035	-0.369, 0.299	-0.039	0.837
R^2	0.117			
Moderate PA	0.002	-0.015, 0.019	0.027	0.818
Gender	-0.908	-1.625, -0.190	-0.358	0.014
Age	0.084	-0.153, 0.322	0.123	0.480
Tanner	-0.066	-0.408, 0.277	-0.071	0.703
R^2	0.112			
Vigorous PA	0.015	-0.013, 0.042	0.123	0.303
Gender	-0.872	-1.567, -0.177	-0.359	0.015
Age	0.081	-0.149, 0.311	0.124	0.484
Tanner	-0.068	-0.398, 0.262	-0.77	0.681
R^2	0.112			
MVPA	0.003	-0.008, 0.014	0.063	0.547
Gender	-0.976	-1.638, -0.315	-0.416	0.004
Age	0.103	-0.114, 0.320	0.164	0.347
Tanner	-0.114	-0.427, 0.198	-0.135	0.468
R^2	0.140			
